# Transformation of sperm structure in *Octopus vulgaris*: From spermatogenesis to spermatophoric release

**DOI:** 10.1371/journal.pone.0316519

**Published:** 2025-01-22

**Authors:** Hyeon Jin Kim, Jung Jun Park, Jung Sick Lee

**Affiliations:** 1 Department of Aqualife Medicine, Chonnam National University, Yeosu, Republic of Korea; 2 Aquaculture Industry Research Division, East Sea Fisheries Research Institute, National Institute of Fisheries Science, Gangneung, Republic of Korea; Zhejiang University College of Life Sciences, CHINA

## Abstract

The present study describes the differentiation process of male germ cells in *Octopus vulgaris*, the morphology of sperm in the testis and spermatophore, and the sperm released after the spermatophoric reaction. During spermatogenesis, the male sperm cell gradually elongates from a round shape, with cytoplasm shifting toward the head and the acrosome forming. Additionally, in the spermatid stage, the flagellum develops within the posterior nuclear channel and extends outside the cytoplasm. The sperm is composed of a head and a tail. The head is approximately 17.9 μm long and consists of a highly electron-dense nucleus and a helical acrosome. The tail is divided into three parts: the mid-piece, principal-piece, and end-piece. The mid-piece forms a mitochondrial sheath with 7–8 mitochondria surrounding a “9+2” axoneme. The principal-piece is composed of an axoneme, outer dense fibers, and fibrous sheath, while the end piece lacks outer dense fibers or fibrous sheath. The sperm in the testis and spermatophore, and the sperm released after the spermatophoric reaction have the same structure. However, in the sperm located in the testis and spermatophore, the structure of the acrosome is unclear due to the presence of cytoplasm in the head. In contrast, sperm released after the spermatophoric reaction lack their cytoplasm, revealing the helical acrosome. This unique sperm morphology, adapted for internal fertilization, is thought to be advantageous for fertilization and long-term storage within the female reproductive system.

## 1. Introduction

In animals, fertilization can be either external or internal, depending on the fertilization site. Although fertilization is predominantly external in aquatic animals, some species, including chondrichthyans [1], some teleosts [2–4], crustaceans [5], and cephalopods [6], exhibit internal fertilization. Regardless of the fertilization site, gametogenesis is a critical prerequisite for successful reproduction. Gametogenesis is the process by which gametes differentiate and develop through meiosis [7, 8]. Spermiogenesis, a part of this process, involves morphological changes in spermatids, such as the formation of the acrosome and tail, chromatin condensation in the karyoplasm, and shedding of cytoplasm.

Sperm morphology and ultrastructure vary widely depending on species and animal phyla [9]. Ultrastructural studies of sperm have been reported in octopods such as *Bathypolypus bairdii* and *B*. *sponsalis* [10, 11], *Opisthoteuthis persephone* [12], *Octopus minor* [13–15], *O*. *ocellatus* [14, 16], *O*. *tankahkeei* [17, 18], and *Vulcanoctopus hydrothermalis* [11].

Spermatogenesis and sperm morphology not only differ between species, but also vary depending on reproductive strategies [19]. For example, *Hynobius leechii* [20], *Neoditrema ransonneti* [3], crustaceans [5], and cephalopods [21] have specific strategies to transfer sperm to females in the form of spermatophores. These spermatophores are released from the funnel when delivered to the female, and sperm are released in the form of spermatangium when a spermatophoric reaction is triggered by a pulling stimulus [6].

*O*. *vulgaris* is a species distributed worldwide and the dominant species among cephalopods in Korea. Like other octopuses, it exhibits reproductive ecological characteristics such as sexual dimorphism, spermatophore formation, and sperm storage in females after mating. Unlike squid and cuttlefish, *O*. *vulgaris* carry out internal fertilization [6]. To understand the mechanism of sperm storage within the female reproductive organs, it is essential to first describe the ultrastructure of the sperm. Studies on the sperm ultrastructural differentiation of *O*. *vulgaris* have only been reported for acrosomal morphogenesis [22, 23], and chromatin organization during spermiogenesis [24]. Therefore, in this study, we investigated the microstructural differentiation of the sperm of *O*. *vulgaris* and compared the structure of sperm in the testis and spermatophore with that of sperm after a spermatophoric reaction. Through these structural analyses, we aim to understand the reproductive strategies of this species and provide basic data on reproductive ecology.

## 2. Materials and methods

### 2.1. Specimens

In this study, adult male *Octopus vulgaris* (mean total weight 434.8 ± 294.1 g, n = 295) were used for analysis. The specimens were collected using octopus pots in Yeosu (N34°35’, E127°43’) on the southern coast of South Korea, from May 2022 to July 2023.

### 2.2. Light microscopy

Light microscopy was performed according to the methodology previously described [25]. *O*. *vulgaris* were dissected, and testis were fixed in aqueous Bouin’s solution, rinsed in running water, and dehydrated with an ethanol series (70%-100%). Sections were prepared according to the paraffin method using a microtome (Leica, Wetzlar, Germany). Samples were treated with Mayer’s hematoxylin-0.5% eosin (H-E) stain, Masson’s trichrome stain, alcian blue-periodic acid and Schiff’s solution (AB-PAS, pH 2.5) reaction.

### 2.3. Scanning electron microscopy

A portion of the testis was excised and prefixed in 2.5% glutaraldehyde buffered with 0.1 M phosphate (pH 7.4) for 3 hours. The fixed samples were washed three times for 20 minutes each with 0.1 M phosphate buffer (pH 7.4), dehydrated in an ascending series of alcohols, and then dried. Afterwards, the surface was coated with platinum (10 mA/4 min) using a metal ion coater (SC7620, Quorum, UK) and observed using FE-SEM (Sigma 500, Zeiss, Germany) at an acceleration voltage of 1 kV.

The sperm in the spermatophores were analyzed using a method modified from the one described above. For analysis of sperm before spermatophoric reaction, embedded spermatophore specimens were sectioned into 3 μm thick sections using a microtome (Leica, Wetzlar, Germany). Then, the sections were placed on aluminum plates, paraffin was removed using xylene and the sections were dried, coated with platinum ions, and then observed. Sperm after the spermatophoric reaction was observed in the same way as the testis after inducing a spermatophoric reaction to make the sperm mass into a suspension.

### 2.4. Transmission electron microscopy

Samples for transmission electron microscopy were prefixed in 2.5% glutaraldehyde buffered with 0.1 M phosphate (pH 7.4), followed by post-fixation with 1% osmium tetroxide at 4°C. After fixation, the samples were washed with 0.1 M phosphate buffer (pH 7.4) and dehydrated using ethanol. The samples were then embedded in an epoxy resin using the substitution process with propylene oxide. The finished epoxy block was sectioned to a 60 nm thickness using an ultramicrotome (EM-UC 7, Leica, Germany), double-stained with uranyl acetate-lead citrate, and observed using FE-SEM (Sigma 500, Zeiss, Germany).

### 2.5. Image analysis

Histological quantification of male germ cell was performed by analyzing 10 to 15 cells at each stage. Microscope images were converted into JPEG files and analyzed using an image analyzer (i-Solution, IMT Inc., USA). The size of the cells was measured as the minor axis and the major axis, and the ratio of nucleus to cytoplasm of the germ cell was calculated as follows.


$$Proportion\;of\;nucleus\;to\;cytoplasm\;(\%)=\frac{Nucleus\;area\;({㎛}^{2})}{Cytoplasm\;area\;({㎛}^{2})}\times 100$$

## 3. Results and discussion

### 3.1. Structure of the testis

**Fig 1:**
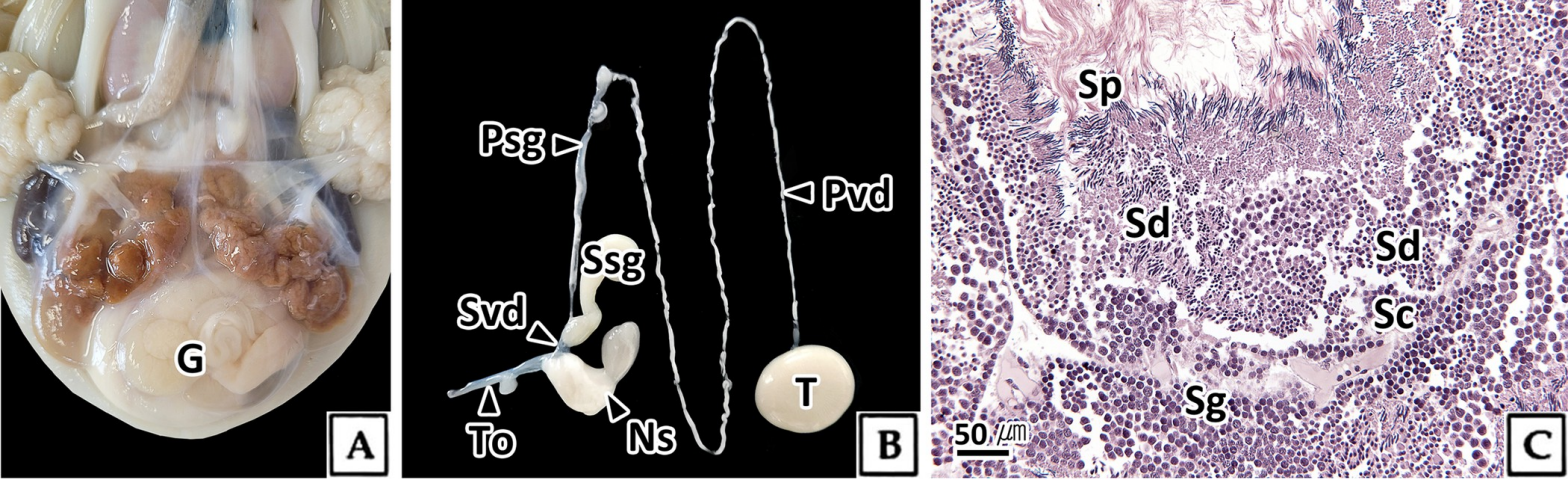
The male reproductive system (A, B) and testis structure (C) in *Octopus vulgaris*. C: light microscopy, H-E stain. G: gonad, Ns: Needham’s sac, Psg: primary spermatophoric gland, Pvd: primary vas deferens, Sc: spermatocytes, Sd: spermatids, Sg: spermatogonia, Sp: sperm, Ssg: secondary spermatophoric gland, Svd: secondary vas deferens, T: testis.

The testis of adult *O*. *vulgaris* was milky white and circular (Fig 1A and 1B). Histologically, it was of the seminiferous tubule type, consisting of numerous tubules, and the seminiferous tubules were composed of thin connective tissue. Within the seminiferous tubules, spermatogonia, spermatocytes, spermatids, and sperm were arranged in a layered order from the cortex to the medulla (Fig 1C).

### 3.2. Spermatogenesis

Spermatogenesis in *O*. *vulgaris* was divided into four stages, spermatogonium, spermatocyte, spermatid, and sperm, based on cell size, stainability, and structural features of the nucleus and organelles.

#### 3.2.1. Spermatogonium

**Fig 2:**
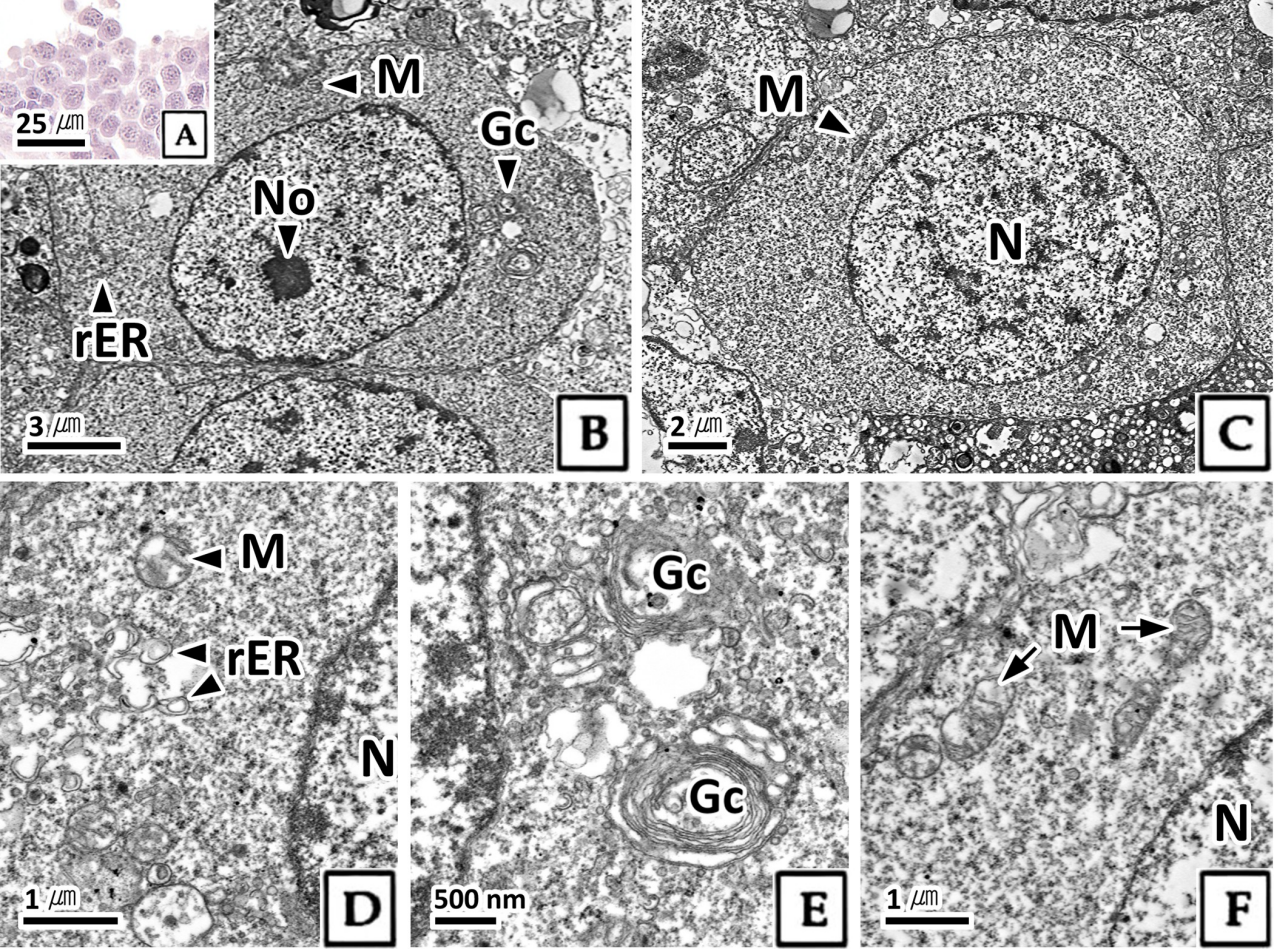
Photomicrographs of spermatogonium in *Octopus vulgaris*. A: light microscopy, H-E stain. B-F: transmission electron microscopy. B: interphase. C: multiplicative stage. Gc: Golgi complex, M: mitochondria, N: nucleus, No: nucleolus, rER: rough endoplasmic reticulum.

The spermatogonium was oval and approximately 10.1 μm in diameter (Table 1). The karyoplasm exhibited strong basophilic chromatin in the H-E stain, while the cytoplasm displayed weak basophilia (Fig 2A). In transmission electron microscopy, spermatogonia in the interphase appeared oval-shaped and the nucleus was occupying most of the cell. The karyoplasm was filled with highly electron-dense nucleolus and heterochromatin, and less electron-dense euchromatin (Fig 2B). The cytoplasm contained tubular mitochondria, rough endoplasmic reticulum and Golgi complex (Fig 2D and 2E). The nucleus of the spermatogonium in the multiplicative stage was elliptical, occupying more than 60% of the cell, similar to the interphase. In the karyoplasm, condensed heterochromatin and euchromatin were distinguished, and the nucleolus observed in interphase was not observed (Fig 2C). Numerous mitochondria were observed throughout the cytoplasm (Fig 2F).

**Table 1:** Size and the ratio of nucleus to cytoplasm of the male germ cell in spermatogenesis.

Stage	Cell (μm)	Nucleus (μm)	Ratio of N to C (%)
Spermatogonium	10.1±1.0 × 8.0±0.8	7.4±1.0 × 6.8±0.6	60.4±7.3
Spermatocyte	8.9±0.8 × 7.3±0.8	6.8±0.6 × 6.1±0.6	54.8±13.0
Early spermatid	5.4±0.7 × 3.8±0.7	2.9±0.3 × 2.7±0.3	36.1±7.0
Mid spermatid	7.3±1.7 × 3.2±0.7	3.2±0.7 × 2.2±0.3	34.1±4.2
Late spermatid	10.7±3.3 × 3.3±0.4	9.4±1.0 × 1.1±0.1	33.0±4.2

#### 3.2.2. Spermatocyte

**Fig 3:**
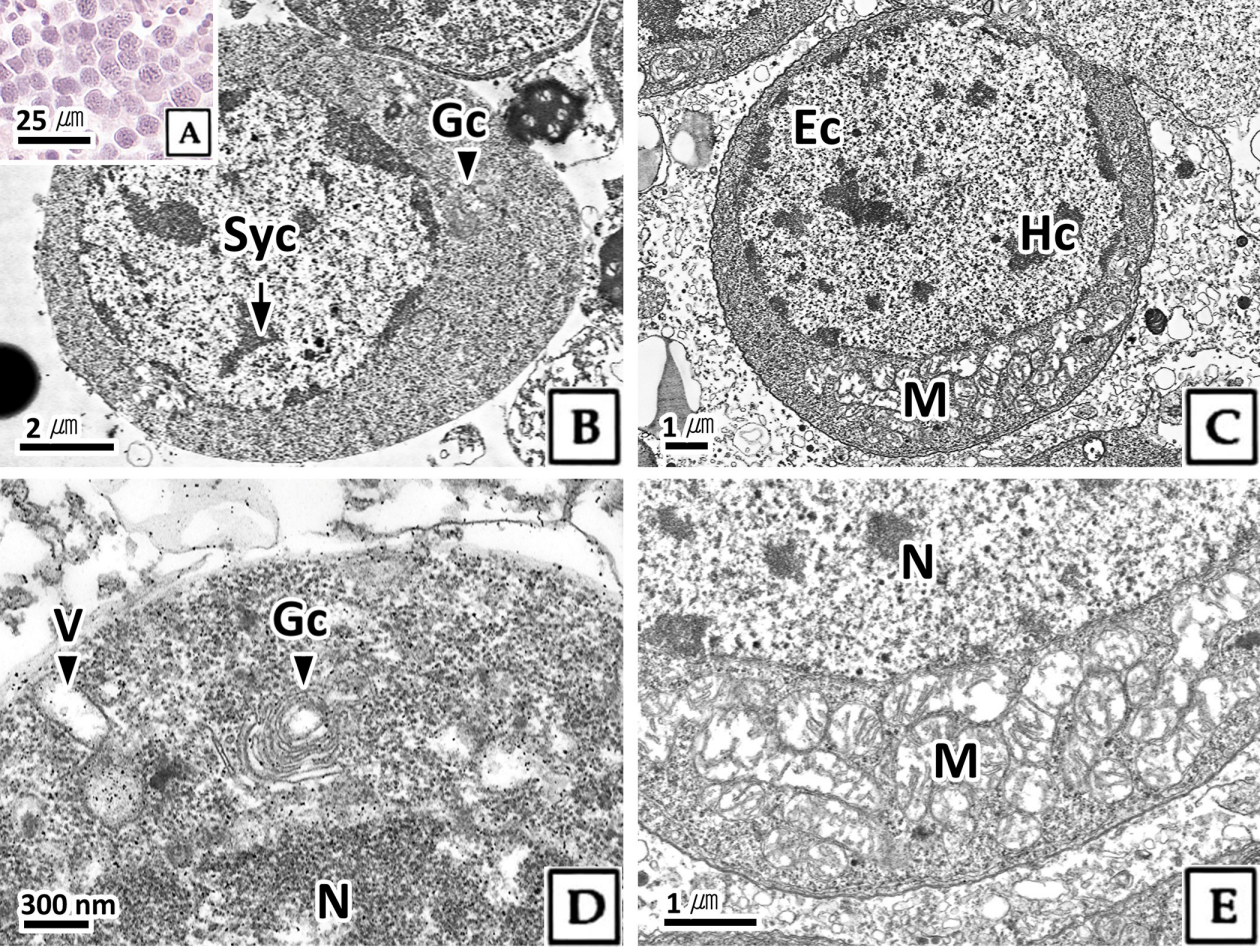
Photomicrographs of spermatocyte in *Octopus vulgaris*. A: light microscopy, H-E stain. B-E: transmission electron microscopy. B: primary spermatocyte showing the synaptonemal complex (Syc) in karyoplasm. C-E: secondary spermatocyte. D: note the Golgi complex (Gc) at the top of nucleus (N), E: note the mitochondria (M) at the basal side of the nucleus. Ec: euchromatin, Hc: heterochromatin, V: vesicle.

The spermatocyte was smaller than the spermatogonium and approximately 8.9 μm in size (Table 1). In H-E stain, strongly basophilic chromosomes were observed within the karyoplasm, while the cytoplasm exhibited weak basophilia (Fig 3A). In transmission electron microscopy, the primary spermatocyte was oval-shaped, similar to a spermatogonium, with some condensed granular heterochromatin within the nucleus and a double-filamentous condensed synaptonemal complex (Fig 3B). In secondary spermatocytes, condensed heterochromatin was clearly visible in the karyoplasm (Fig 3C). A circular Golgi complex with developed cisternae and low electron-dense vesicles was observed in the cytoplasm, located apical to the nucleus (Fig 3D), while numerous mitochondria were distributed basal to the nucleus (Fig 3E).

#### 3.2.3. Spermatid

**Fig 4:**
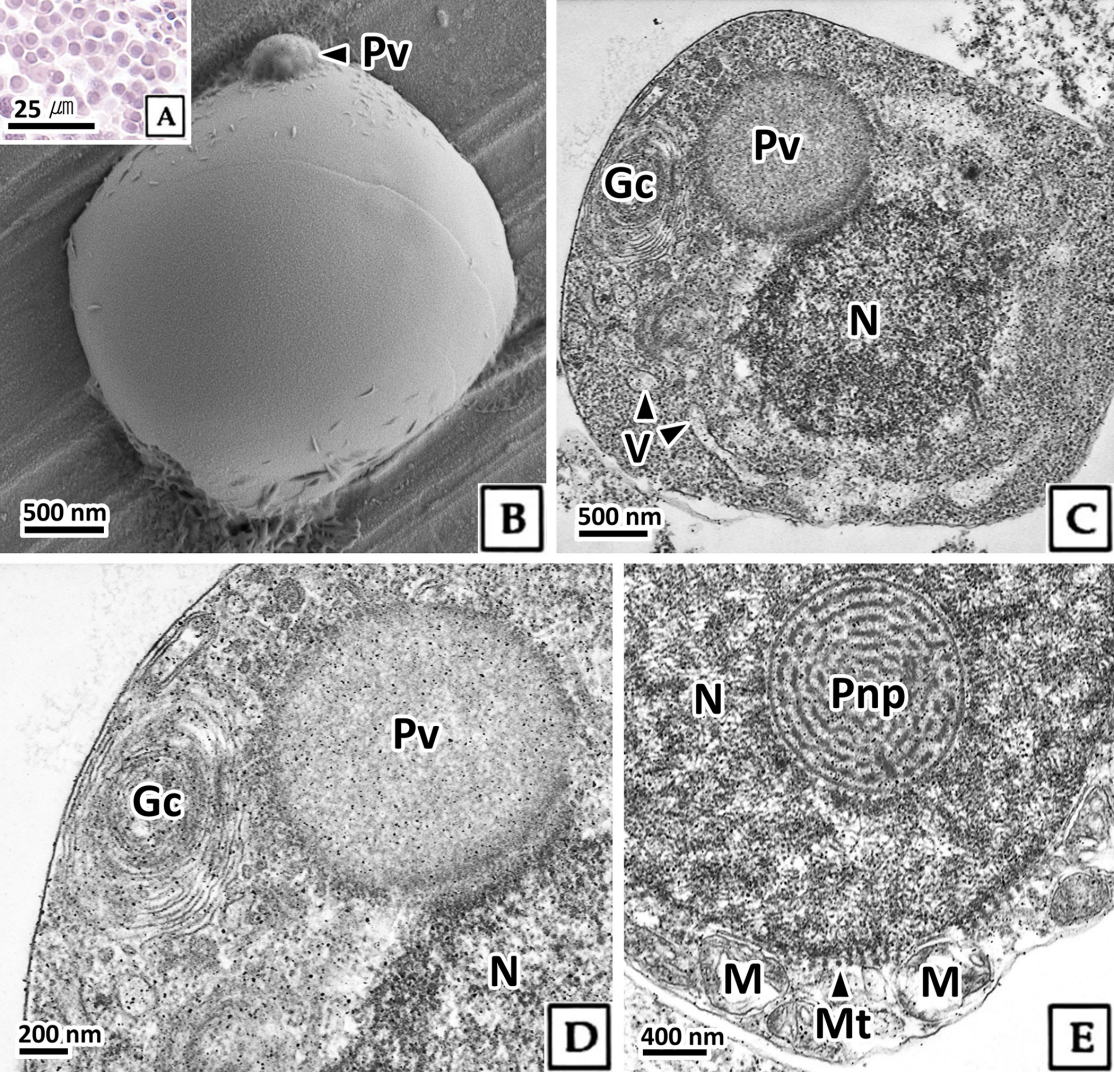
Photomicrographs of early spermatid in *Octopus vulgaris*. A: light microscopy, H-E stain. B: scanning electron microscopy. C-E: transmission electron microscopy. B and C: note the proacrosomal vesicle (Pv) at the apical zone of the nucleus. D: note the well-developed Golgi complex (Gc) with proacrosomal vesicle. E: the posterior nuclear pocket (Pnp) of the nucleoplasm. microtubule (Mt) adhered to nucleus (N), and mitochondria (M) at the basal side of nucleus. V: vesicle.

Based on cell size and the structural features of the nucleus and organelles, the spermatid stage was divided into three phases: early, mid, and late. The early spermatid remained circular but decreased in size compared to the spermatocyte to approximately 5.4 μm (Table 1). The nucleus exhibited strong basophilia in H-E stain, and the ratio of nucleus to cytoplasm was 36.1%, a decrease of 18.7% compared to the previous stage (Fig 4A, Table 1). Scanning electron microscopy showed that as spermatogenesis progressed, cell size decreased, and proacrosomal vesicles appeared in the apical zone of the cells (Fig 4B). In transmission electron microscopy, the karyoplasm of early spermatids was mostly composed of fibrillar heterochromatin (Fig 4C). A circular posterior nuclear pocket with a labyrinth pattern was present at the basal side of the nucleus, and microtubules surrounded the nucleus (Fig 4E). In the cytoplasm, a Golgi complex developed at the apical part of the nucleus, forming a circular proacrosomal vesicle with low electron density (Fig 4D). Numerous mitochondria were densely packed at the basal side of the nucleus (Fig 4E).

**Fig 5:**
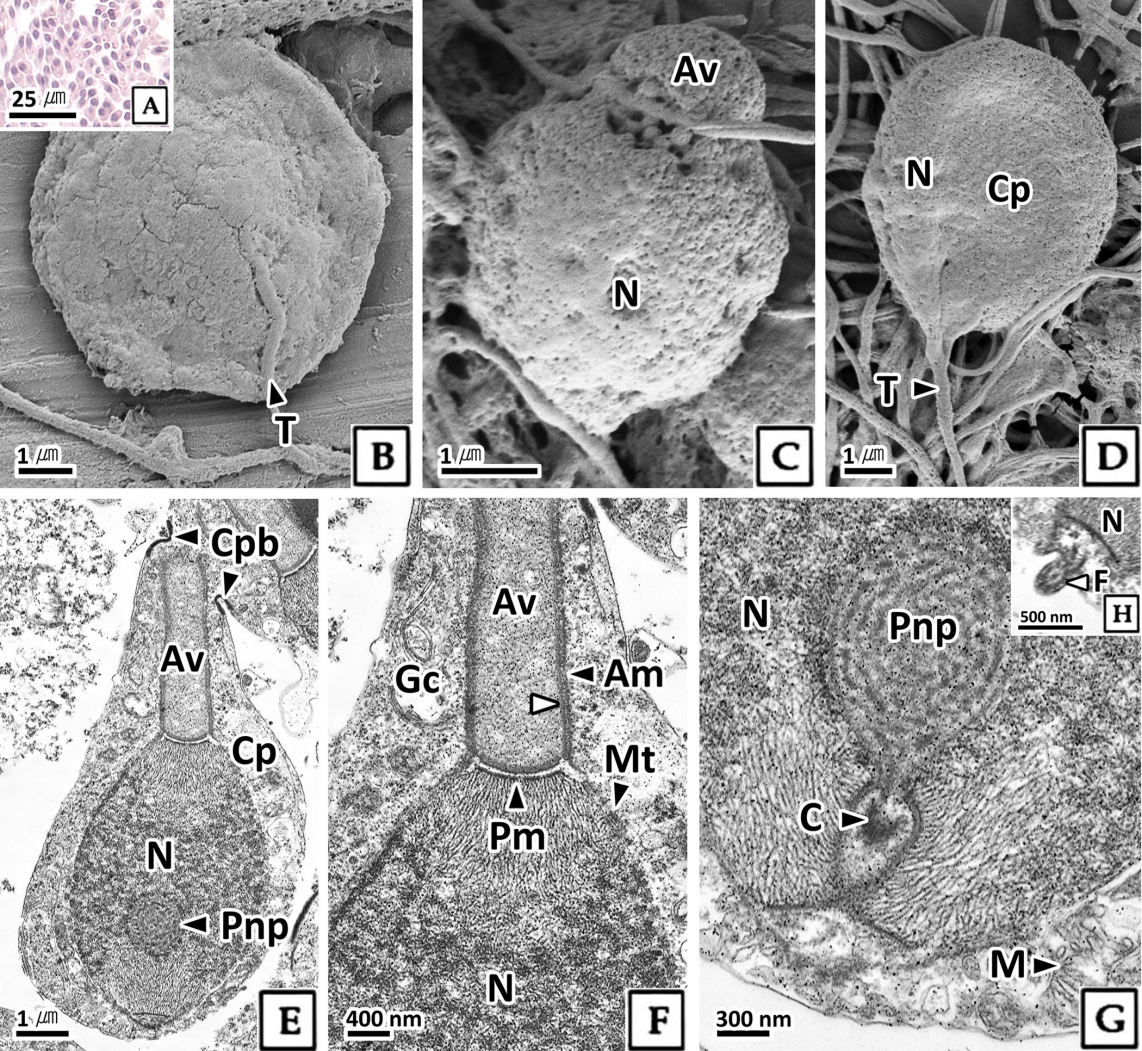
Photomicrographs of mid spermatid in *Octopus vulgaris*. A: light microscopy, H-E stain. B-D: scanning electron microscopy, acrosomal vesicle (Av) and tail (T) formation. E-H: transmission electron microscopy. E: cytoplasmic bridge (Cpb) between two mid spermatids. F: The acrosomal vesicle surrounded by the external (black arrowhead) and internal (white arrowhead) acrosomal membrane (Am), and separated from the nucleus (N) by the plasma membrane (Pm). G and H: centriole (C) and flagellum (F) formed form posterior nuclear pocket (Pnp). Cp: cytoplasm, Gc: Golgi complex, M: mitochondria, Mt: microtubule.

Compared to early spermatids, mid spermatids were elongated, measuring 7.3 μm in length and 3.2 μm in thickness (Fig 5A, Table 1). In scanning electron microscopy, the nucleus was elongated from circular to rectangular shape, and the formation of a tail and acrosome was observed (Fig 5B–5D). By transmission electron microscopy, the karyoplasm of mid spermatids was observed to be mainly composed of fibrillar heterochromatin, with chromatin orientation confirmed toward the apical and basal sides (Fig 5E). In the posterior nuclear pocket located at the lower end of the nucleus, a basal body composed of centrioles was formed, and a flagellum developed from the basal body protruding from the cytoplasm (Fig 5G and 5H). In the cytoplasm, a developed Golgi complex at the apical side of the nucleus, and elongated acrosomal vesicles were present. The acrosomal vesicle consisted of two layers and was separated from the nucleus by the plasma membrane (Fig 5F). Numerous mitochondria were densely packed at the basal side of the nucleus (Fig 5G).

**Fig 6:**
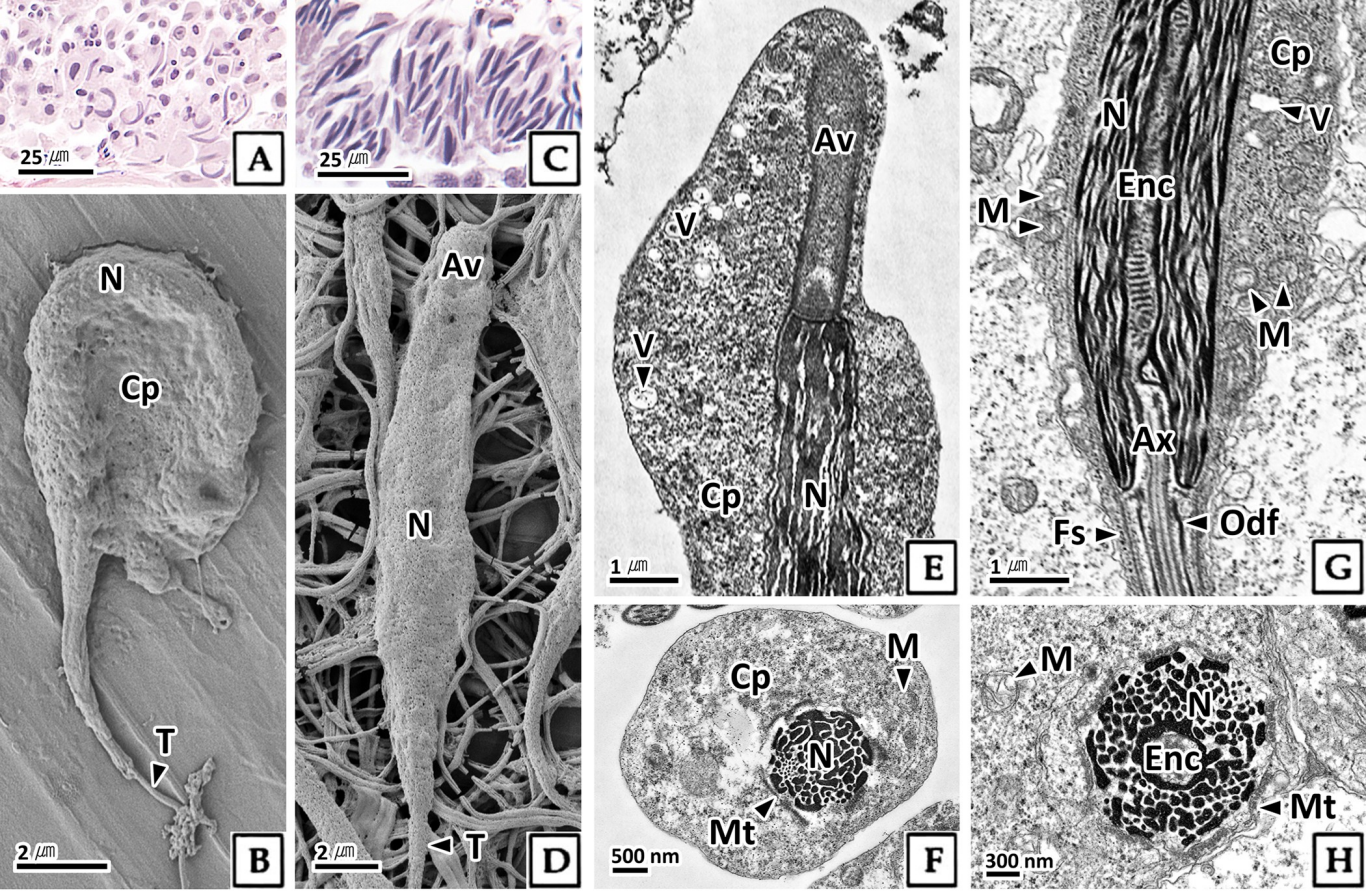
Photomicrographs of late spermatid in *Octopus vulgaris*. A and C: light microscopy, H-E stain. B and D: scanning electron microscopy. E-H: transmission electron microscopy. E: anterior part of the late spermatid, note the large number of vesicles (V) within the cytoplasm (Cp). F: cross section of the anterior nucleus (N). G: posterior part of the late spermatid, the endonuclear channel (Enc) of the nucleus connects to the axoneme (Ax). H: cross section of the posterior nucleus, and microtubule (Mt) adhered to nucleus. Av: acrosomal vesicle, Fs: fibrous sheath, M: mitochondria, Odf: outer dense fibers, T: tail.

Compared to mid spermatids, late spermatids were longer (10.7 μm in length and 3.3 μm in thickness) (Table 1), and their nuclei changed from a curved to a straight shape (Fig 6A–6D). In transmission electron microscopy, the karyoplasm of late spermatids increased in size due to the fusion of condensed fibrillar heterochromatin (Fig 6E and 6G). In the central part of the nucleus, the posterior nuclear pocket elongated and transformed into an endonuclear channel, which connected to the axoneme of the tail (Fig 6G). In cross sections, the periphery of the nucleus was encircled by microtubules (Fig 6F and 6H). The acrosomal vesicle was elongated and bullet-shaped compared to that of mid spermatids, and the cytoplasm shifted to the upper part of the cell. Numerous vesicles were present in the upper part of the cytoplasm (Fig 6E), and round mitochondria were distributed in the lower part (Fig 6G and 6H).

#### 3.2.4. Sperm

**Fig 7:**
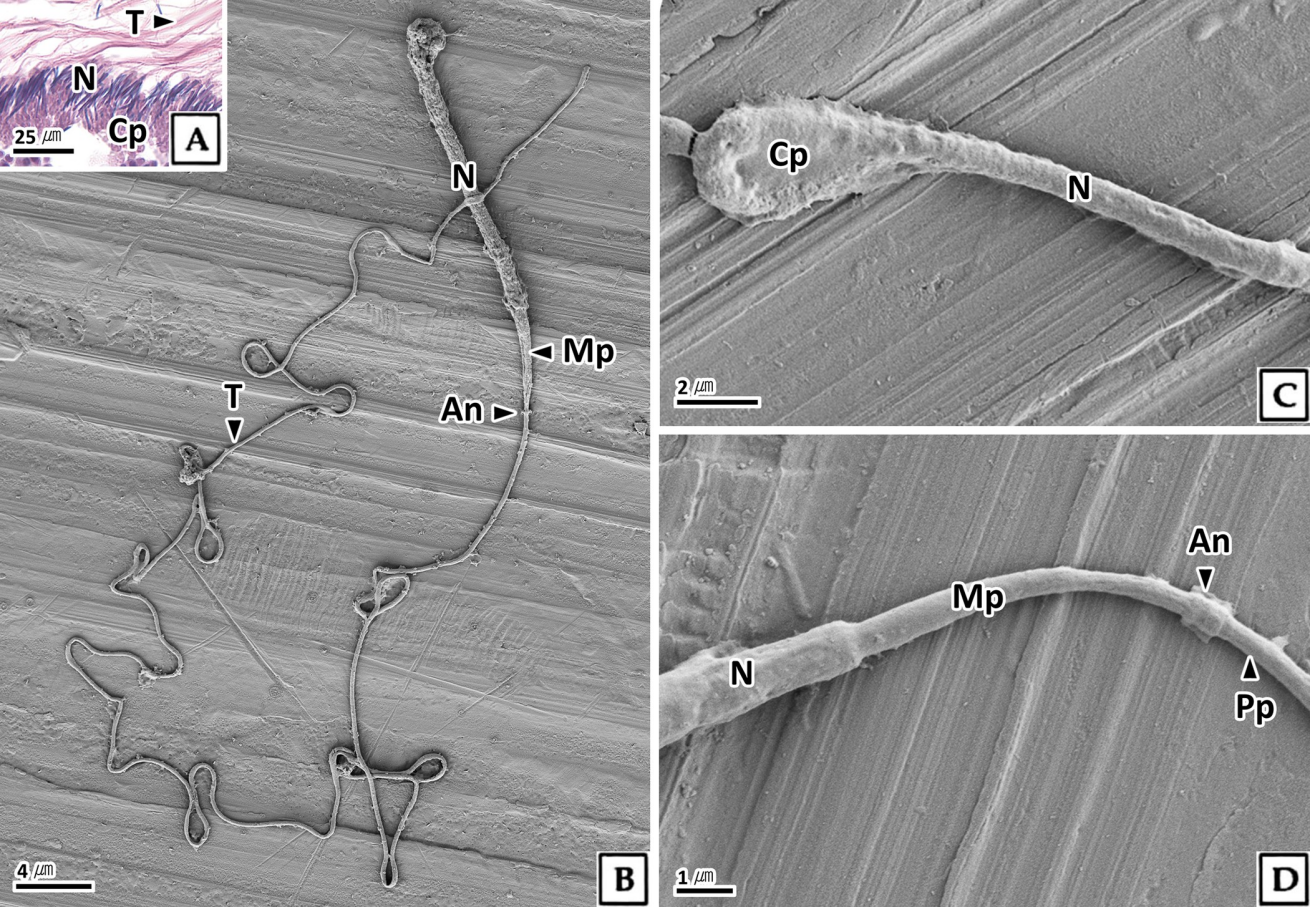
Photomicrographs of sperm in the testis of *Octopus vulgaris*. A: light microscopy, H-E stain. B-D: scanning electron microscopy. B: sperm morphology. C: head. D: showing annulus (An) between the mid-piece (Mp) and principal-piece (Pp). Cp: cytoplasm, N: nucleus, T: tail.

The sperm in the testis were approximately 215.3 μm in total length and consists of a head and a tail (Fig 7A and 7B). The sperm head measured 16.3 μm in length and 0.9 μm in diameter, was rectangular with a rounded end, consisted of a nucleus and an acrosome, and was covered with cytoplasm (Fig 7C). In H-E stain, the cytoplasm and tail showed eosinophilic reactions, while the nucleus stained strongly basophilic (Fig 7A). The tail was single and about 199.1 μm long, and an annulus was identified between the mid-piece and the principal-piece via scanning electron microscopy (Fig 7B and 7D).

**Fig 8:**
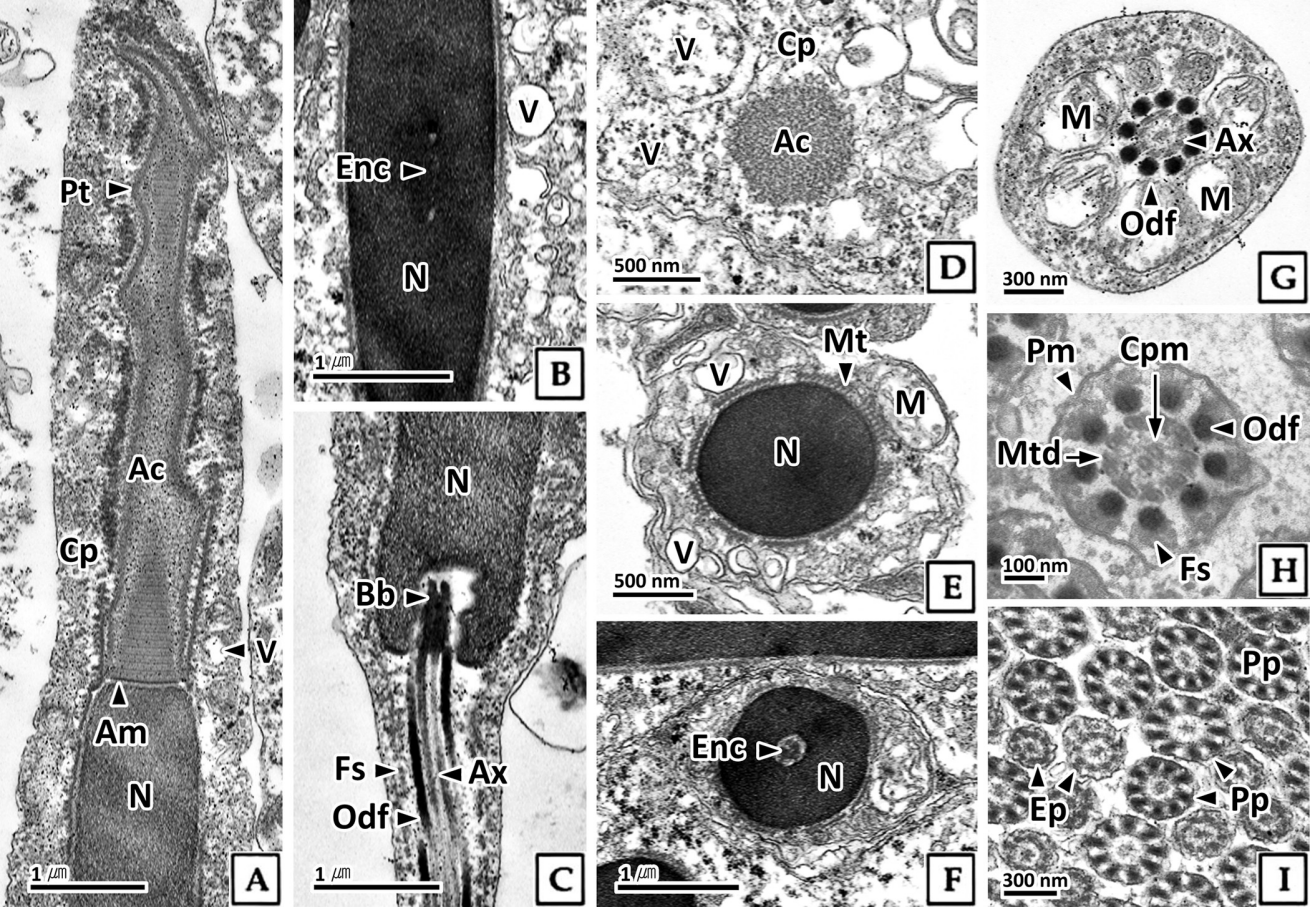
Transmission electron microscopy of the sperm in the testis of *Octopus vulgaris*. A-C: longitudinal section, D-I: cross section. A, D, E: anterior part of head. B and F: mid part of head. C: posterior part of head. G: mid-piece, showing the mitochondria (M) surrounding axoneme (Ax). H: principal-piece (Pp), showing the plasma membrane (Pm), fibrous sheath (Fs), outer dense fibers (Odf), microtubule doublets (Mtd), central pair microtubule (Cpm). I: principal-piece and end-piece (Ep). Ac: acrosome, Am: acrosomal membrane, Bb: basal body, Cp: cytoplasm, Enc: endonuclear channel, Mt: microtubule, N: nucleus, Pt: protuberance, V: vesicle.

Sperm in the testis observed by transmission electron microscopy were surrounded by microtubules (Fig 8). The head contained a helical acrosome with stripes surrounded by an acrosomal membrane, and its electron density was lower than that of the nucleus (Fig 8A and 8D). The nucleus was rectangular, with electron-dense and homogeneous karyoplasm, and an endonuclear channel located in the center of the nucleus (Fig 8B and 8F). Numerous vesicles were identified in the cytoplasm of the head (Fig 8B and 8E).

The sperm tail was divided into a mid-piece, principal-piece, and end-piece. The mid-piece was composed of a mitochondrial sheath containing 7–8 mitochondria surrounding the axoneme (Fig 8G). The principal-piece consisted of an axoneme, outer dense fibers, and a fibrous sheath (Fig 8H), whereas the end-piece lacked outer dense fibers and the fibrous sheath (Fig 8I). The axoneme displayed a "9+2" structure with two central microtubules surrounded by nine pairs of microtubule doublets. The outer dense fibers were composed of nine fibers and were the most electron-dense structures. The fibrous sheath was absent in the mid-piece and end-piece, and the granules in the principal-piece were less electron-dense than the outer dense fibers (Fig 8C and 8H).

**Fig 9:**
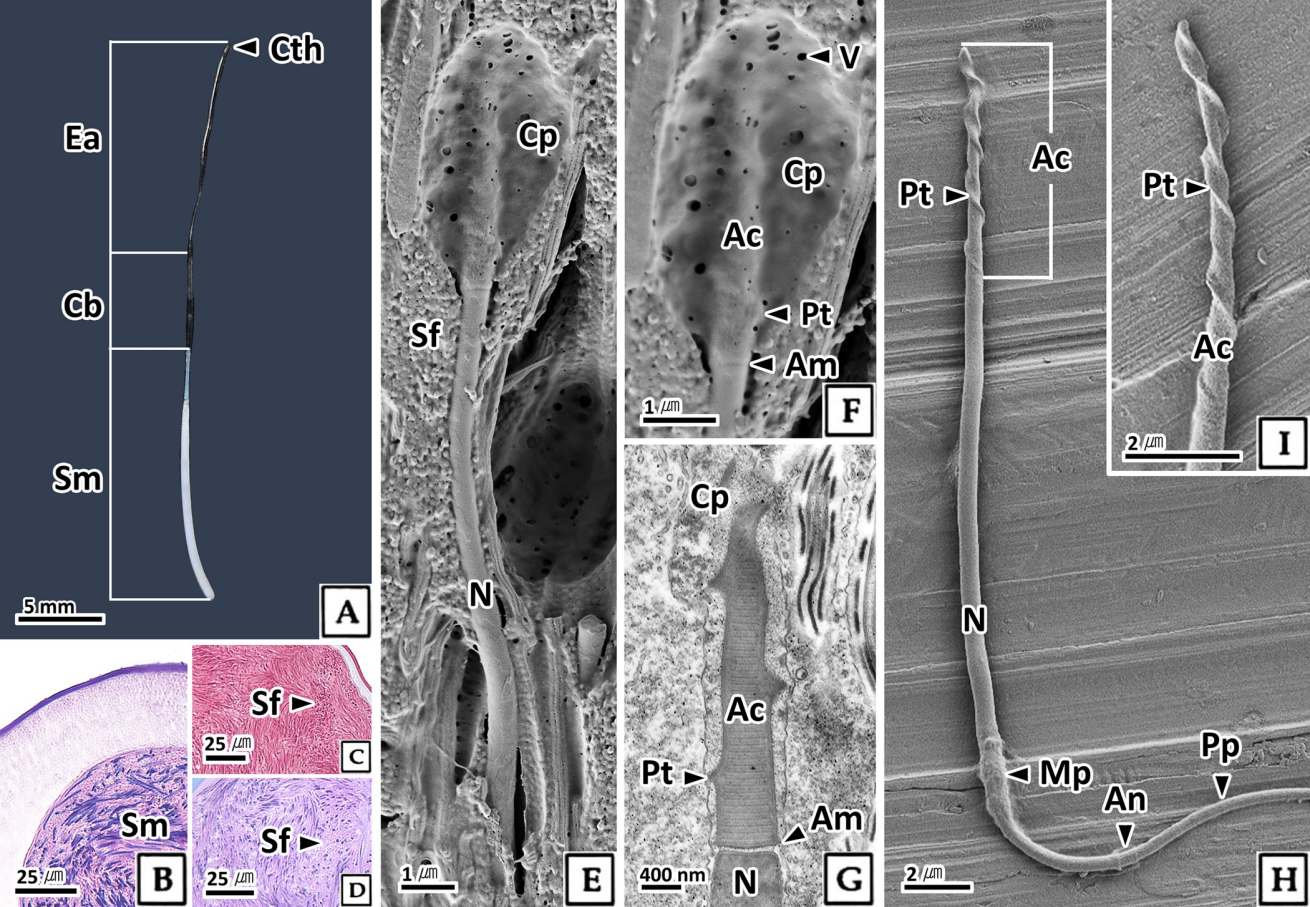
Photomicrographs of the sperm within the spermatophore (A) and released sperm after the spermatophoric reaction in *Octopus vulgaris*. B-D: light microscopy, sperm mass (Sm) of spermatophore. B: H-E stain. C: Masson’s trichrome stain. D: AB-PAS (pH 2.5) reaction. E, F, H and I: scanning electron microscopy. G: transmission electron microscopy. E-G: sperm in the spermatophore before spermatophoric reaction, note that the acrosome (Ac) is surrounded by cytoplasm (Cp). H and I: Sperm released from the spermatophore by spermatophoric reaction. Am: acrosomal membrane, An: annulus, Cb: cement body, Cth: cap thread, Ea: ejaculatory apparatus, Mp: mid-piece, N: nucleus, Pp: principal-piece, Pt: protuberance, Sf: spermatophoric fluid, V: vesicle.

Sperm formed in the testis passes through the primary vas deferens, primary and secondary spermatophoric glands to form spermatophore, and the formed spermatophore passes through the secondary vas deferens to be stored in the Needham’s sac (Fig 1B). The spermatophore consists of a cap thread, ejaculatory apparatus, cement body, and sperm mass (Fig 9A), and the sperm within the sperm mass exhibits a concentric structure (Fig 9B). The sperm within the spermatophore was surrounded by the spermatophoric fluid, a spherical substance. The spermatophoric fluid appeared eosinophilic in H-E stain, red in Masson’s trichrome stain, and reacted with PAS to appear reddish purple in the AB-PAS (pH 2.5) reaction, indicating that it was composed of neutral carboxylated mucopolysaccharides (Fig 9B–9D). The acrosome of the sperm head was surrounded by cytoplasm containing numerous vesicles measuring 4.9 μm in length and 2.6 μm in thickness, similar to sperm in the testis (Fig 9E–9G). However, the sperm released from the spermatophore after the spermatophoric reaction were different in that the cytoplasm was completely removed (Fig 9H and 9I). Scanning electron microscopy revealed that the sperm head was approximately 19.5 μm in length, with an acrosome length of approximately 7.3 μm and a diameter of about 0.4 μm, exhibiting a distinct helical structure. A mid-piece was located at the rear of the nucleus, and an annulus was identified between the mid-piece and the principal-piece (Fig 9H and 9I).

## 4. Discussion

### 4.1. Spermatogenesis

**Fig 10:**
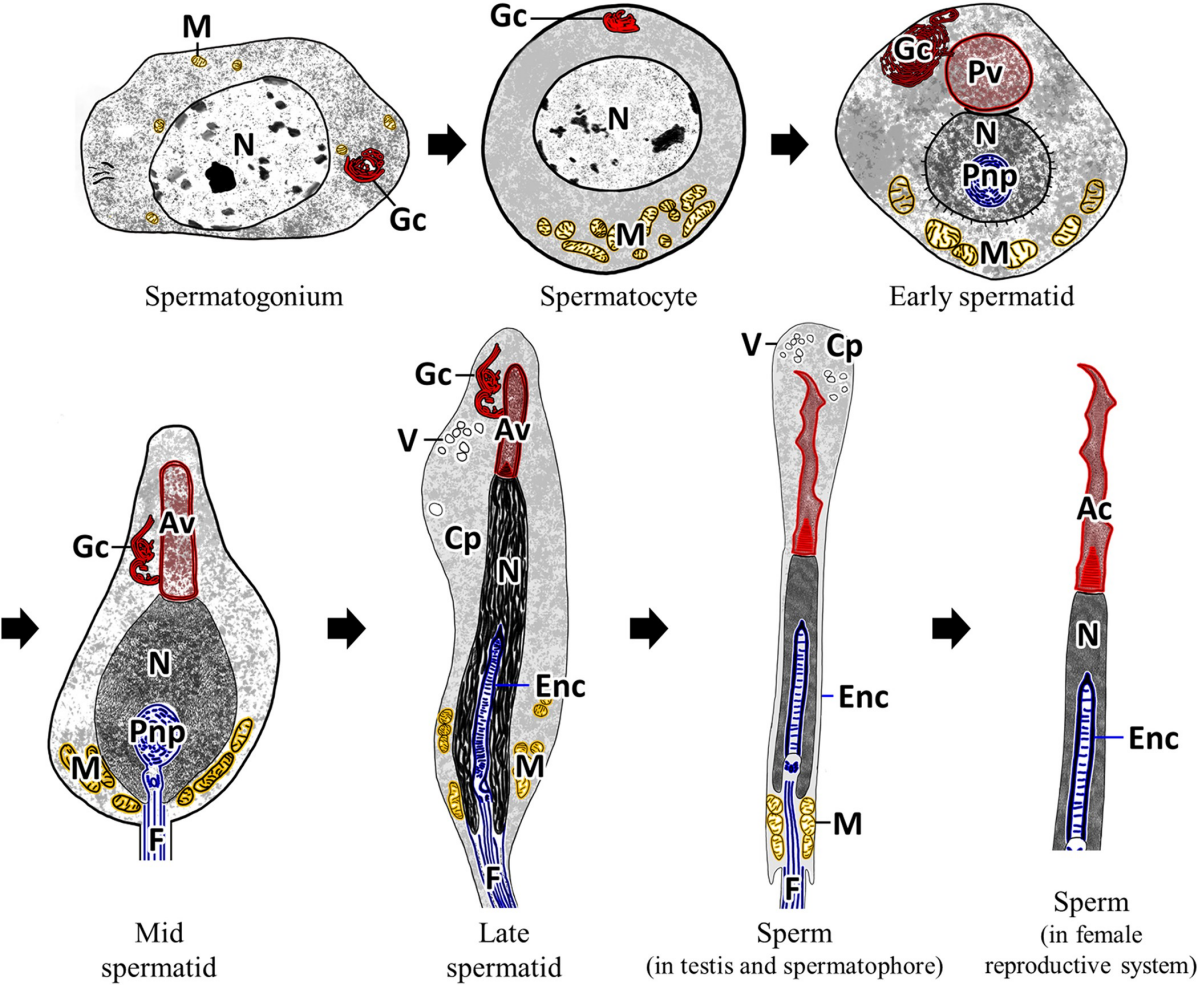
Diagram of male germ cell at each stage during spermatogenesis in *Octopus vulgaris*. Ac: acrosome, Av: acrosomal vesicle, Cp: cytoplasm, Enc: endonuclear channel, F: flagellum, Gc: Golgi complex, M: mitochondria, N: nucleus, Pnp: posterior nuclear pocket, Pv: proacrosomal vesicle, V: vesicle.

Spermatogenesis consists of spermatogonium, spermatocyte, spermatid, and sperm stages [26]. In the present study, spermatogenesis in *O*. *vulgaris* was divided into these four stages based on morphological changes such as cell size, shape, and organelle (Fig 10).

The most important changes in spermatogenesis are the appearance of the synaptonemal complex, morphological changes in the nucleus, and acrosome and flagellum formation [27, 28]. The formation of synaptonemal complexes is limited to the prophase of first meiosis and is the process by which chromatin dispersed within the nucleus assembles into homologous chromosomes to proceed with meiosis. In this study, the synaptonemal complex was observed, which can be used as an important characteristic to distinguish primary spermatocytes from secondary spermatocytes [29].

The acrosome plays an important role in the process of sperm penetration into the oocyte. The acrosome is formed when a proacrosomal granule formed in the Golgi complex develops into an acrosomal vesicle. In general, the activation of these Golgi complexes and the formation of acrosomal vesicles occur at the secondary spermatocyte or early spermatid stage [30]. In this study, proacrosomal vesicles of *O*. *vulgaris* were formed in early spermatids, and acrosomal vesicles were formed in mid spermatids.

Changes in cell morphology mainly include chromatin condensation in the karyoplasm and shedding of the cytoplasm and karyoplasmic condensation is classified into a granular pattern, fibrillar pattern, and lamellar pattern [31]. The karyoplasmic condensation occurs due to gradual changes in the interactions between DNA and associated proteins, resulting in a decrease in nucleus volume [24]. In cephalopods, various karyoplasmic condensation patterns have been reported. In *Sepia officinalis*, there was a change from a fibro-granular structure to fibro-type [32]. In *Spirula spirula*, as spermiogenesis progresses, coarsely granular patches condense into a cylindrical, homogeneous state through lateral fusion via a fine fibrous reticulum, and mature sperm within the spermatophore are uniformly electron dense [33]. Among octopods, in *E*. *cirrhosa*, karyoplasmic condensation results in the formation of fibers about 10 nm in diameter, and nuclear condensation continues with the formation of thicker fibers which tend to aggregate at the nuclear periphery [34]. These results are similar to those previously reported in *O*. *tankahkeei* [35]. Ribes et al. reported the chromatin condensation pattern of *O*. *vulgaris* to be fibrotubular [24]. However, in the case of *O*. *minor*, it is initially granular, but changes to slender and fibrous within the chromatin, and then aggregates and becomes homogenous [15]. In this study, *O*. *vulgaris* had a karyoplasmic condensation pattern of granular heterochromatin in spermatocytes and fibrillar chromosomes in early and mid-spermatids. At the end of spermatid differentiation, chromatin was judged to be condensed into a single homogeneous state through a polymerization process, corresponding to a fibrillar type condensation pattern.

During spermiogenesis, the nucleus in mid and late spermatids was surrounded by microtubules (manchettes). These features have also been shown in *V*. *infernalis* [36], *E*. *cirrhosa* [34], and *O*. *tankahkeei* [35]. The manchette, composed of laterally associated microtubules, appears transiently during spermatogenesis [37]. It is involved in nucleus formation, including movement and remodeling of spermatid chromatin [38].

In *O*. *vulgaris*, the changes associated with flagellar formation occur in three stages. 1) In the early spermatid, a rounded posterior nuclear pocket appears, encircling the nuclear rod. 2) In the mid spermatids, a centriole forms in the posterior nuclear pocket, and the flagellum is released from the cytoplasm. 3) In the late spermatid, this posterior nuclear pocket elongates and transforms into an endonuclear channel. The endonuclear channel, called the extra-nuclear rod, nuclear fossa, fibrous plug, or implantation fossa, is found in the karyoplasm of spermatids in octopods [10, 11, 22, 36, 39] and is a type of centriolar rootlet composed of centrioles [36]. The mechanism of flagellum formation observed in this study aligns with the processes reported for *O*. *minor* [13] and *O*. *tankahkeei* [40].

### 4.2. The structure of sperm

Sperm exhibit various characteristics depending on biological classification criteria. Based on the fertilization site, sperm are classified into aquasperm and introsperm, and based on the presence or absence of an acrosome, they are categorized as acrosomal sperm or anacrosomal sperm [41]. Most aquatic animals, except teleosts, possess acrosomes, the shape and size of which vary across species [42]. The acrosome of *O*. *vulgaris* [22] and *Eledone cirrhosa* [34] appears as a spherical or sac-shaped vesicle. The acrosome length was reported to be approximately 7.1 μm in *O*. *ocellatus* [16], 5.5 μm in *O*. *minor* [15], and 5 μm in *O*. *tankahkeei* [17], all exhibiting a single helical structure. In contrast, *B*. *bairdii* and *B*. *sponsalis* have acrosome lengths of 8.57 μm and 8.17 μm, respectively, with a double helical keel structure [10]. In octopods, the acrosome is a helical structure, surrounded by cytoplasm in an acrosomal vesicle space or lacuna between the acrosome and the cell membrane. In this study, sperm in the testis and spermatophore of *O*. *vulgaris* were surrounded by cytoplasm containing multiple vesicles with a single helical acrosome, although cytoplasm was not observed in sperm after the spermatophoric reaction.

The removal of cytoplasm during spermatogenesis streamlines the sperm, facilitating efficient swimming [43]. It has been reported that water pumps are involved in the cytoplasm removal of *O*. *tankahkeei* sperm [35]. In this study, it was hypothesized that the cytoplasm in *O*. *vulgaris* sperm is removed through vesicles, reducing hydrodynamic resistance and improving motility as the sperm move from the spermatophore to the spermathecae in the female reproductive system.

The length, shape, and acrosomal structure of the sperm head are closely associated with the structure of the oocyte membrane [44]. In octopods, the specific function of the acrosome has not yet been fully elucidated because sperm penetrate the oocyte through the micropyle [45]. After copulation, sperm are known to pass through the oviduct and are stored in the spermathecae of the oviducal gland, where they attach to the epithelial layer of the spermathecae, and the acrosome is likely to play a mechanical role in sperm storage [46, 47]. In this study, it was observed that the helical acrosome structure of the sperm functions as an anchoring mechanism, embedding the sperm within the epithelial layer of the female oviduct. However, further studies are needed to confirm the structural configuration and state of stored sperm within the female reproductive organs.

Sperm motility is crucial for reproduction in sexually reproducing organisms and is driven by the mechanical movement of the axoneme, the structure of which varies by species. Typically, the tail of the sperm is composed of 9+2 microtubular axonemes. The tail is divided into the mid-piece, principal-piece, and end-piece, with the mid-piece comprising mitochondria arranged around the axoneme [48]. In cephalopods, the shape of the mid-piece varies among taxa, including forms such as a mitochondrial spur, a sleeve-like mid-piece, or a mitochondrial sheath [33, 34, 36, 49]. In this study, *O*. *vulgaris* was found to have 7–8 mitochondria surrounding the axoneme, arranged in a row in the longitudinal section, indicating the presence of a mitochondrial sheath. The mitochondrial sheath is an adaptation found in sperm involved in internal fertilization, allowing for long-term movement within the female reproductive tract and storage of the energy required for fertilization [50].

The annulus is a septin-based ring structure located at the junction of the mid-piece and the principal-piece of the flagella of spermatozoa [51]. Scanning electron microscopy revealed an annulus posterior to the mid-piece of *O*. *vulgaris* sperm. This structure has also been observed in octopods such as *O*. *bimaculatus* [39], *Eledone* [52], *B*. *bairdii*, and *B*. *sponsalis* [10], as well as in mammalian sperm, where the annulus acts as a barrier to protein diffusion and helps regulate the correct organization of the mid-piece [48, 53].

In most aquatic animals, the sperm tail consists of an axoneme surrounded by a plasma membrane. In octopods such as *B*. *bairdii* and *B*. *sponsalis* [10], *O*. *ocellatus* [16], *O*. *tankahkeei* [17], and *O*. *minor* [15], the principal-piece consists of outer dense fibers and fibrous sheaths in addition to the axoneme. In this study, *O*. *vulgaris* exhibited the same structure. Outer dense fibers and fibrous sheaths are mainly observed in species that undergo internal fertilization, such as mammals and amniotes [54, 55]. Unlike sperm from externally fertilizing species that swim in water, sperm from internally fertilizing species must pass through the viscoelastic fluids of the female reproductive organ. It has been proposed that this structural adaptation such as outer dense fibers and fibrous sheaths inhibits the buckling of the axoneme in such environments [56, 57].

The morphological changes and structural features of sperm that occur during spermatogenesis in *O*. *vulgaris* were analyzed. The sperm of *O*. *vulgaris* display morphological characteristics closely associated with sperm storage and fertilization, distinguishing them from the primitive sperm of cephalopods. These differences are thought to be structural adaptations for internal fertilization and long-term storage within the female reproductive system. These results highlight the functional importance of sperm in the reproductive strategies of *O*. *vulgaris* and provide essential data on its reproductive physiology. This study described the structure of sperm before they enter the female reproductive system. However, there has been little research on how their structure changes once inside the female reproductive system, so further investigation into sperm structure within the female reproductive system is necessary.
